# A qPCR Method to Assay Endonuclease Activity of Cas9-sgRNA Ribonucleoprotein Complexes

**DOI:** 10.4014/jmb.2305.05010

**Published:** 2023-06-19

**Authors:** Minh Tri Nguyen, Seul-Ah Kim, Ya-Yun Cheng, Sung Hoon Hong, Yong-Su Jin, Nam Soo Han

**Affiliations:** 1 Brain Korea 21 Center for Bio-Health Industry, Division of Animal, Horticultural, and Food Science, Chungbuk National University, Cheongju 28644, Republic of Korea; 2 Department of Food Science and Human Nutrition, University of Illinois at Urbana-Champaign, Urbana, IL 61801, USA; 3 Carl R. Woese Institute for Genomic Biology, University of Illinois at Urbana-Champaign, Urbana, IL 61801, USA; 4 Faculty of Biology, Dalat University, 01- Phu Dong Thien Vuong, Dalat, Vietnam

**Keywords:** CRISPR-Cas9, Cas9 activity, Cas9 ribonucleoprotein complex, kinetics of Cas9, qPCR

## Abstract

The CRISPR-Cas system has emerged as the most efficient genome editing technique for a wide range of cells. Delivery of the Cas9-sgRNA ribonucleoprotein complex (Cas9 RNP) has gained popularity. The objective of this study was to develop a quantitative polymerase chain reaction (qPCR)-based assay to quantify the double-strand break reaction mediated by Cas9 RNP. To accomplish this, the dextransucrase gene (*dsr*) from *Leuconostoc citreum* was selected as the target DNA. The Cas9 protein was produced using recombinant *Escherichia coli* BL21, and two sgRNAs were synthesized through in vitro transcription to facilitate binding with the *dsr* gene. Under optimized in vitro conditions, the 2.6 kb *dsr* DNA was specifically cleaved into 1.1 and 1.5 kb fragments by both Cas9-sgRNA365 and Cas9-sgRNA433. By monitoring changes in *dsr* concentration using qPCR, the endonuclease activities of the two Cas9 RNPs were measured, and their efficiencies were compared. Specifically, the specific activities of *dsr*365RNP and *dsr*433RNP were 28.74 and 34.48 (unit/μg RNP), respectively. The versatility of this method was also verified using different target genes, uracil phosphoribosyl transferase (*upp*) gene, of *Bifidobacterium bifidum* and specific sgRNAs. The assay method was also utilized to determine the impact of high electrical field on Cas9 RNP activity during an efficient electroporation process. Overall, the results demonstrated that the qPCR-based method is an effective tool for measuring the endonuclease activity of Cas9 RNP.

## Introduction

Clustered regularly interspaced short palindromic repeats (CRISPR) and their associated proteins (Cas) are adaptive immune systems of prokaryotes against foreign DNA, including phages, viruses, and plasmids [1, 2]. The breakdown of foreign DNA is mediated by Cas nucleases, which are site-specific cleavage enzymes of invading nucleic acids [3]. The advantage of this nuclease-based system is that the target DNA sequence is recognized by simple base-pairing complementarity using sgRNA [4]. Recently, the CRISPR-Cas9 system has been developed as a versatile genome-editing tool that can be widely used for modifying the genomes of both prokaryotic and eukaryotic organisms. This system can change chromosomal targets through insertion, deletion, replacement, and silencing in various organisms [5, 6].

For the delivery of Cas9 to cells, a Cas9-sgRNA ribonucleoprotein complex (Cas9 RNP) is often used owing to its fast DNA cleavage activity, ease of preparation, and low off-target effects [7]. Exogenously delivered Cas9 RNP does not rely on transcriptional or translational cellular machinery nor carries any long nucleic acids or viral-based approaches that could integrate into the genome [8]. Furthermore, Cas9 RNP does not involve DNA; thus, no undesirable DNA footprints are left in the host genome [9]. For these reasons, the delivery of Cas9 with sgRNA in the form of a Cas9 RNP complex has been applied to various organisms, including animals, plants, fungi, yeast, and microalgae [10]. The endonuclease activity of Cas9 depends on various factors, including the structure of Cas9 and sgRNA, the ratio of sgRNA and Cas9, and the presence of cofactors and inhibitors [11, 12]. For the efficient application of Cas9 RNP in genome editing, measuring the endonuclease activities of Cas9 consisting of different sgRNAs remains challenging.

In general, the endonuclease activity of a Cas9 can be monitored by various methods, including electrochemiluminescence [13], fluorescence dye such as Tao/IowaBlack [14], and radiolabeling with g-^32^P or 6-FAM on target DNA [15, 16]. However, electrochemiluminescence or fluorescence methods require [Ru(bpy)_3_]^2+^ or fluorescent labeling on the target DNA sequence, and radioactive signals pose a severe health risk for researchers [17]. These methods are complex, time-consuming, risky, and unstable for labeled fluorescence. Therefore, there is an urgent need to develop a simple, stable, and highly efficient method for monitoring Cas9 endonuclease activity.

Quantitative or real-time polymerase chain reaction (qPCR) is a powerful tool for simultaneously detecting or quantifying the concentration of a targeted DNA by employing a PCR-based technique that enables the enumeration of the copy number of genes in a complex DNA sample [18-20]. The primary principle of qPCR is to measure the fluorescent signal produced by dyes, including SYBR green, which binds to double-stranded DNA [21]. Double-stranded breaks (DSB) induced by Cas9 RNP decrease the target DNA concentration, resulting in a reduced fluorescent signal, allowing quantification of the target DNA using qPCR.

In this context, we measured the Cas9 endonuclease activity in vitro using quantitative PCR. The dextransucrase gene (*dsr*) of *Leuconostoc citreum* EFEL2700 [22] was amplified using PCR, and the amplicon was used as the target DNA to be cleaved by Cas9 RNP. The Cas9 protein was prepared using recombinant *Escherichia coli* BL21 cells, and the two sgRNAs (sgRNA365 and sgRNA433), which guide the binding of Cas9 RNP with *dsr* DNA, were prepared using in vitro transcription. Quantitative PCR was conducted using the primer sets which bind to either side of *dsr*, and the *dsr* amplicon concentration was measured using a standard plot. Next, the endonuclease activities of the Cas9 were calculated based on the amplicon concentration changes, and their efficiencies were compared. Furthermore, to further verify the applicability of qPCR method for monitoring Cas9 activity across diverse target DNA, uracil phosphoribosyl transferase (*upp*) gene of *Bifidobacterium bifidum* was used as target DNA for DSB induction using Cas9 RNP. Finally, the assay method developed in this study was applied to investigate the effect of a pulsed electrical field on the activity of Cas9 RNP for an efficient electroporation process.

## Materials and Methods

### Bacterial Strain, Plasmid, and Growth Conditions

The strain used in this study was *Leuconostoc citreum* EFEL2700 (EFEL2700) (former depository number, CB2567) grown in De Man, Rogosa, and Sharpe (MRS, USA) medium at 30°C and *Bifidobacterium bifidum* BGN4 grown in MRS medium containing 0.05% L-cysteine∙HCl at 37°C. *E. coli* BL21 (DE3) was used for protein overproduction. The plasmid pET28a-Cas9-His was obtained from Addgene, Inc. (USA). The recombinant *E. coli* BL21 strain harboring plasmid pET28a-Cas9-His was grown in Luria–Bertani (LB) broth (BD Biosciences, USA) at 37°C with shaking at 200 rpm, and the transformants were selected on LB agar medium supplemented with 50 μg/ml kanamycin [23].

### Preparation of Dextransucrase Gene amplicon (*dsr*) as Target DNA

For genomic DNA (gDNA) extraction, L. citreum EFEL2700 was grown in MRS medium at 30°C under anaerobic conditions to 1 × 10^9^ CFU/ml. Cells were centrifuged at 10,000 ×*g* for 1 min, and chromosomal DNA was extracted from cell pellets using a genomic DNA (gDNA) prep kit (Solgent, Korea). The *dsr* gene of EFEL2700 was amplified by PCR using chromosomal DNA as a template. With extracted gDNA, PCR was carried out using 20 μl of reaction mixture containing 2.0 μl of Ex Taq buffer, 2.0 μl of 10 mM dNTP, 1.0 μl of 10 pM each primer DSU-F and DSU-R, 12.8 μl of sterile distilled water, 1 μl of template DNA (100 -150 ng/μl), and 0.2 μl of 0.2 U/μl Ex Taq polymerase (Takara, Japan). For *upp* gene of *B. bifidum*, UPP-F and UPP-R were used as primers (Table 1). A MiniAmp Plus Thermal Cycler (Applied Biosystems, USA) was used for PCR, and the amplification conditions were as follows: one cycle at 94°C for 5 min; 30 cycles at 94°C for 1 min; annealing at 58°C for 20 s; elongation at 72°C for 2 min; and one cycle at 72°C for 5 min. The *dsr* and *upp* amplicon were purified using the AccuPrep PCR/Gel Purification Kit (Bioneer, Korea), and DNA concentration and purity were measured using a NanoDrop system (Molecular Devices, USA).

### Expression and Purification of Recombinant Cas9 Protein

*E. coli* BL21 cells harboring pET28a-Cas9-His were cultured in LB broth containing 50 μg/ml kanamycin at 37°C with shaking at 200 rpm. When the OD_600_ reached 0.4–0.5, isopropyl β-d-1-thiogalactopyranoside (final concentration 0.8 mM) was added for induction, and additional culture was conducted at 18°C for 12 h. After centrifugation, the cell pellets were resuspended in 10 mL lysis buffer (50 mM NaH_2_PO_4_, 300 mM NaCl, 10 mM imidazole, and distilled water) and lysed with 60 cycles of 5 s of sonication followed by a 5 s pause using Sonics Vibra cell VCX500 (Sonics, USA). The Cas9 protein was purified using a Ni-NTA column (Thermo Fisher Scientific, USA). The purified Cas9 protein was analyzed using sodium dodecyl-sulfate–polyacrylamide gel electrophoresis (SDS-PAGE), and the protein concentration was measured using a BCA protein assay kit (Thermo Fisher Scientific).

### Designing and in vitro Transcription of sgRNA

The sgRNAs targeting *dsr* and *upp* gene were designed using CRISPOR (http://crispor.tefor.net/crispor.py). The DNA template was synthesized by assembling two oligomers with 23 nucleotide overhangs using a MiniAmp Plus Thermal Cycler (Applied Biosystems). PCR was carried out using 50 μl of reaction mixture containing 10.0 μl of 5X HF buffer, 2.5 μl of 10 mM dNTPs, 1.0 μl of 10 pM each primer F (DS365/DS433 or UPP182 /UPP212), and primer R (common) (Table 1), 35.0 μl of sterile distilled water, and 0.5 μl of 0.02 U/μl of Phusion DNA polymerase (Thermo Fisher Scientific). The PCR conditions were as follows: one cycle at 98°C for 10 s, 30 cycles at 98°C for 10 s, annealing at 54°C for 20 s, extension at 72°C for 20 s, and one cycle at 72°C for 5 min. After template extension, the target DNA was purified using a PCR purification kit (Bioneer) following the manufacturer’s instructions. The in vitro transcription reaction was conducted using T7 RNA polymerase following Yu’s protocol, with minor modifications [23]. A mixture (100 μl) containing 10 μl of 10x T7 RNA buffer (40 mM Tris-HCl, 6 mM MgCl_2_, 1 mM DTT, 2 mM spermidine, pH 7.9), 28 μl of 100 mM MgCl_2_, 4.0 μl of 100 mM rNTPs, 2.5 μl of 40.0 U/μl RNase inhibitor, 3 μl template DNA with 500 ng/μl, 47.5 μl of sterile distilled water, and 5.0 μl of 50 U/μl T7 RNA polymerase (New England BioLabs, USA) was reacted at 37°C for 4 h. The resulting sgRNA was purified using an RNA extraction kit (New England BioLabs) following the manufacturer’s instructions. The purified sgRNA was used immediately for the DNA cleavage assay or frozen at –70°C until further use.

### In vitro Endonuclease Cleavage Reaction of Cas9

The Cas9 reaction was performed in a mixture (100 μl) containing 10 μl of 10 nM Cas9 protein, 10 μl of 10 nM sgRNA, 10 μl of 10x NEB buffer 3.1 (100 mM NaCl, 50 mM Tris-HCl, 10 nM MgCl_2_, 100 μg/ml BSA, pH 7.9) (New England BioLabs), and 40 μl sterile distilled water. The mixture was pre-incubated at 37°C for 10 min to allow Cas9 RNPs complexes formation. Next, 30 μl of target DNA (25 nM) was added to the mixture sample at 37°C. Samples collected at different time points were heated at 90°C for five minutes to stop the reaction. The reaction products were analyzed by 1.5% agarose gel electrophoresis or used as qPCR DNA templates.

### Quantitative Polymerase Chain Reaction (qPCR)

The qPCR for the quantification of target substrate DNA during the RNPs reaction was performed in a final volume of 20 μl of the reaction mixture, containing 10 μl of 2x GreenStar qPCR master mix (SYBR Green I Dye, 1 U/μl hot-start DNA polymerase, 250 μM dNTPs, 40 mM Tris-HCl, 60 mM KCl, and 1.5 mM MgCl_2_), 0.5 μl of 80x ROX dye (Bioneer), 3.5 μl of sterile distilled water, 5 μl of template DNA and 0.5 μl of 10 pM each primer q-DSU-F and q-DSU-R or q-UPP-F and q-UPP-R (Table 1). Amplification was carried out at the following temperature profiles: one cycle at 95°C for 5 min, 39 cycles at 95°C for 15 s, and 58°C for 30 s. PCR amplification was followed by melting curve analysis, in which the temperature was decreased from 95°C for 15 s to 65°C for 20 s at a rate of 0.1°C/s, with a continuous collection of fluorescence data using the Run Exicycler 96 Real-time system (Bioneer). All samples were analyzed in triplicate. The experimental data were analyzed using Analysis Exicycler 96 software (Bioneer).

### The Cas9 RNP Activity Assay

To measure the activity of Cas9, the products formed or substrates used for a given period should be measured [24]. In this study, the endonuclease activity of Cas9 was analyzed by measuring the changes in substrate molecules during the cleavage reaction of Cas9 using qPCR. A standard plot for qPCR was established by serial dilution of amplicon DNA against the C_t_ values obtained using real-time PCR. The Cas9 reaction mixture was heated at 90°C for five minutes to stop the reaction and was used as DNA templates for qPCR. The substrate concentration remaining after the reaction was calculated from the C_t_ value of qPCR. One unit enzyme activity was defined as the amount of enzyme required to cleave one nanomole of *dsr* substrate per minute. The catalytic reaction rate of Cas9 RNP was calculated based on the initial enzyme reaction rate at the start of the reaction (*t* = 0).

### Effect of Pulsed Electrical Field Treatment on Cas9 RNP

To examine the applicability of the assay method developed in this study, the effect of pulsed electrical field on the activity of Cas9 RNP was analyzed for an efficient electroporation process. The Cas9 protein and sgRNA were mixed with a ratio of 1:1, then preincubated at 37°C for 10 min to allow Cas9 RNPs complexes formation. A total of 100 μl of Cas9 RNPs were transferred to cold electroporation cuvettes with an interelectrode distance of 0.1 cm (Bio-Rad, USA) and placed on ice for 5 min. A pulse was delivered under the following conditions: 25 μF, 400Ω at field strengths of 8 to 12 kV/cm (Bio-Rad). Cas9 RNPs were immediately recovered in a 1 ml tube placed on ice, and the remaining activities of Cas9 RNP were measured.

### Statistical Analysis

All experiments were performed in triplicate. The qPCR results were analyzed using SPSS 12.0 (SPSS Inc., USA). SigmaPlot software was used to plot graphs.

## Results

### Expression of Cas9

**Fig. 1:**
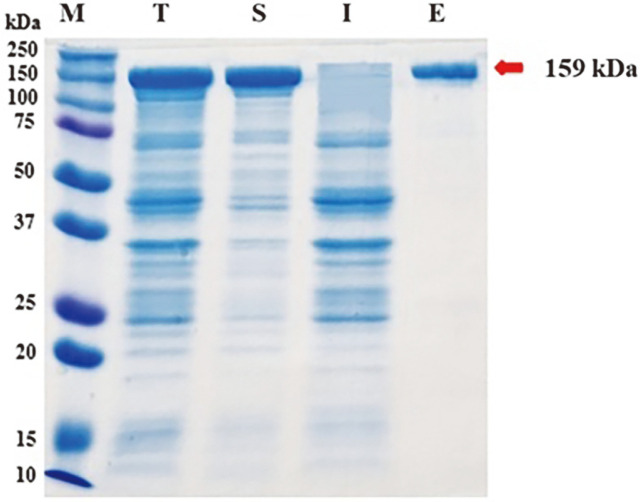
SDS-PAGE Analysis of Cas9 Protein Expressed in *E. coli* BL21. The Cas9 protein, with an approximate molecular weight of 159 kDa, was expressed in *E. coli* BL21 cells that harbored the pET28a-Cas9-His plasmid. The cells were cultivated in Luria Bertani medium. Subsequent purification of the Cas9 protein was achieved using Ni-NTA affinity chromatography.

To obtain an ample supply of Cas9 RNP, Cas9 protein was purified from the culture broth of recombinant *E. coli* BL21 cells containing pET28a-Cas9-His using a Ni-NTA column. The purity and concentration of the purified Cas9 protein were assessed through SDS-PAGE analysis (Fig. 1). The Cas9 protein utilized in this study was derived from *Streptococcus pyogenes* and had a molecular mass of 159 kDa [25]. The SDS-PAGE results indicated that the Cas9 protein appeared as a single band in the 159 kDa region, indicating its successful expression and solubility.

### Transcription of sgRNA

**Fig. 2:**
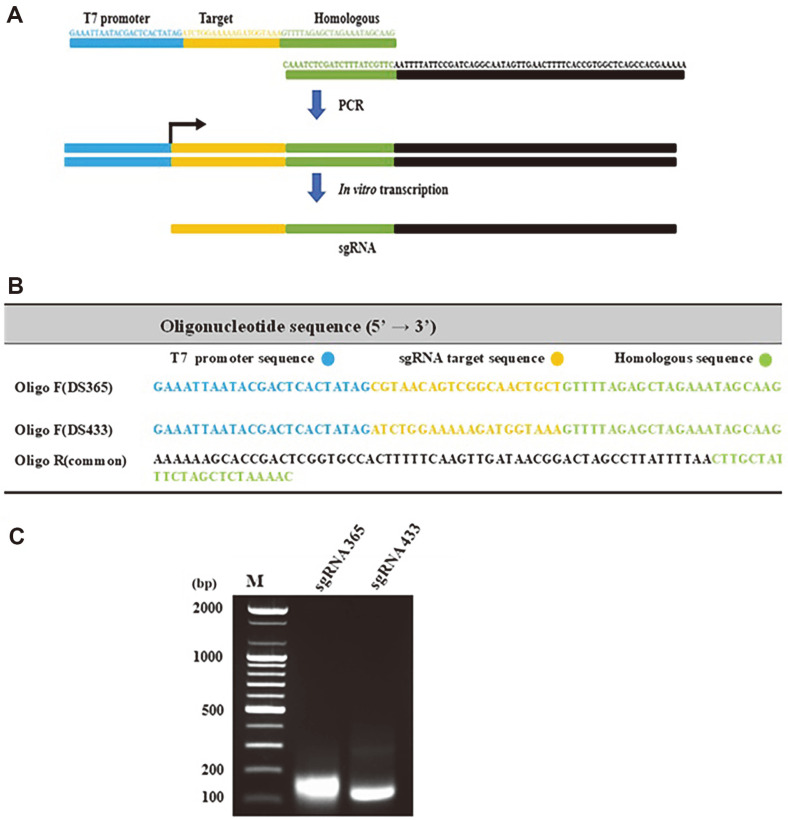
Strategy for synthesizing sgRNA365 and sgRNA433. Both sgRNAs were synthesized by in vitro transcription (**A**), primer sequences of sgRNA (**B**), and agarose gel electrophoresis of T7 RNA polymerase product after transcription of sgRNA365 and sgRNA433 (**C**), Lane M; DNA marker.

The design of sgRNAs targeting the *dsr* and *upp* genes was accomplished using the CRISPOR program [26]. Two sgRNAs, namely DS365 and DS433, were selected from the suggested candidates with high anticipated efficiency rates of 81% and 87%, respectively (Table 2). The DNA template was synthesized by PCR-based assembly of two oligomers with 23 nucleotide overhangs. Subsequently, the two sgRNAs were transcribed in vitro utilizing T7 polymerase, following the procedure illustrated in Fig. 2A, along with the primer set provided in Fig. 2B. As shown in Fig. 2C, the 123-bp bands were successfully amplified in both sgRNAs.

### In Vitro Cas9 Reaction

**Fig. 3:**
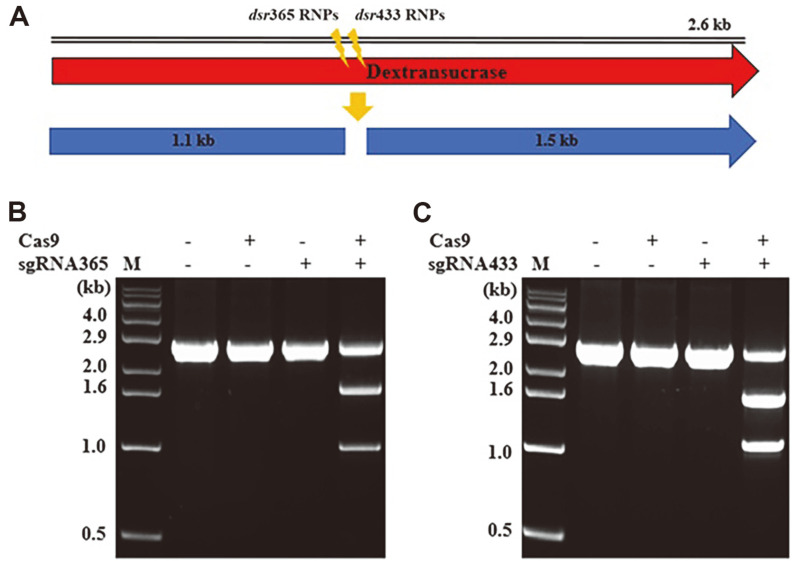
Double-strand break in *dsr* amplicon by *dsr*365 RNP and *dsr*433 RNP. The strategy for the double-strand break in *dsr* amplicon (**A**). Agarose gel electrophoresis results of *dsr* amplicon by *dsr*365 RNP (**B**) and *dsr*433 RNP (**C**). When Cas9 and sgRNAs were mixed, the substrate *dsr* amplicon (2.6 kb) was cleaved to fragment 1 (1.5 kb) and fragment 2 (1.1 kb).

To assess the endonuclease activity of Cas9, each RNP was generated through a mixture of Cas9 and sgRNA, which was then reacted with the *dsr* amplicon (2.6 kb). The results in Fig. 3 showed that both pre-formed *dsr*365 RNP and *dsr*433 RNP cleaved the *dsr* amplicon, producing 1.5 kb and 1.1 kb fragments. This result indicated that the combination of both sgRNA365 and sgRNA433 with Cas9 protein under in vitro conditions yielded *dsr*365 RNP and *dsr*433 RNP, which accurately performed double-strand breaks of the target *dsr* amplicon.

### Optimal Quenching Condition of Cas9 RNP Reaction for qPCR

**Fig. 4:**
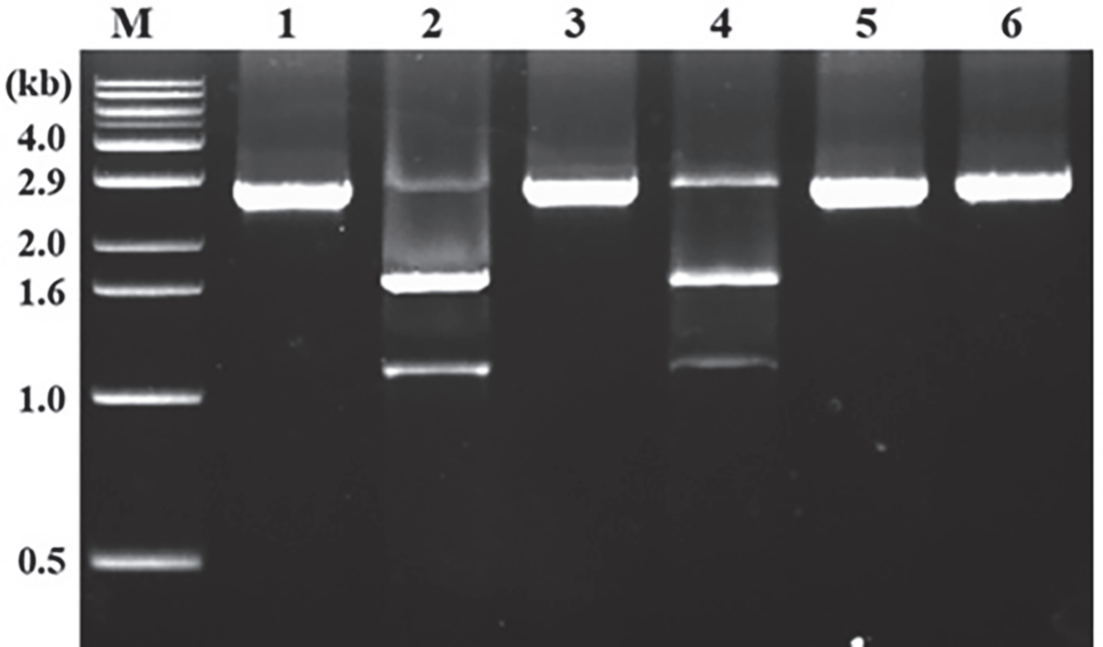
Optimal quenching condition of Cas9 RNPs reaction for qPCR. After Cas9 RNP reaction with *dsr* amplicon at 37°C for 10 min, the reaction was quenched by several methods and analyzed by agarose gel electrophoresis. Lane M; marker for 1 kb, Lane 1, *dsr* substrate; Lane 2, Cas9 RNP, Lane 3, treated by 0.5 M EDTA; Lane 4, treated by 10 U/μl RNase; Lane 5, heating at 90°C for 5 min; Lane 6, treated by 10 U/ μl Protease K.

The catalytic activity of Cas9 needed to be effectively quenched in order to analyze its activity using the qPCR method. As shown in Fig. 4, treatment of Cas9 RNP with RNase enzyme (10 U/μl) failed to completely quench the Cas9 activity, yielding results similar to those of the untreated Cas9 RNP sample. In contrast, complete inhibition of Cas9 RNPs was achieved through treatment with 0.5 M EDTA, heating at 90°C for five minutes, and Protease K (10 U/μl). However, it should be noted that both EDTA and Protease K inhibited the DNA polymerase activity in the qPCR assay [27]. Consequently, the heating method was selected as the optimal condition to effectively quench the Cas9 RNP reaction in all subsequent studies.

### Primer Design and Quantitative PCR

**Fig. 5:**
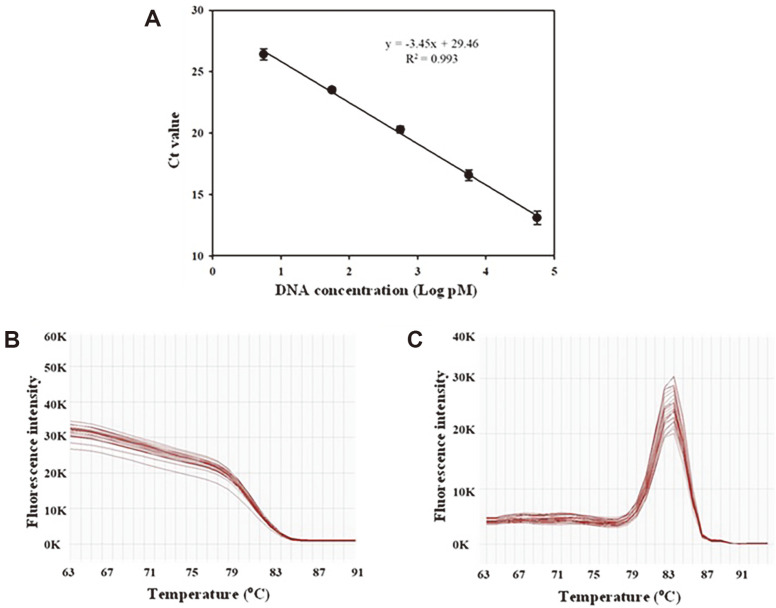
Standard plot for quantification of substrate (*dsr*) concentration (**A**), melting curve (**B**), and melting peak (**C**) analyzed using a real-time polymerase chain reaction. The standard plot was made by plotting the threshold cycle value (C_t_ values) against DNA concentration (Log pM) of *dsr* after serial dilutions of DNA. Data are the average ± standard deviation (SD) of three replicates.

To measure Cas9 endonuclease activity, the concentration of a target DNA was quantified using qPCR using a primer set, q-DSU-F and q-DSU-R (Table 1), which binds to either side of the *dsr* amplicon. The primer set was designed by minimizing the size of the *dsr* amplicon to 183 bp, which is the optimal size for qPCR assays [28]. The standard plot was established by interpolating threshold cycle values (C_t_ values) against the concentration (log pM) of the *dsr* amplicon. As shown in Fig. 5A, the plot showed a -3.45 slope and 0.993 R^2^ value, revealing a high primer efficiency of 95.89% compared with a -3.30 slope of 100% efficiency [20]. In addition, as shown in Fig. 5B and C, the melting curves and peaks exhibited single sharp peaks at 83°C. These results indicated that the primer set could amplify the target gene efficiently.

### Monitoring Endonuclease Activity of Cas9 RNP by qPCR

**Fig. 6:**
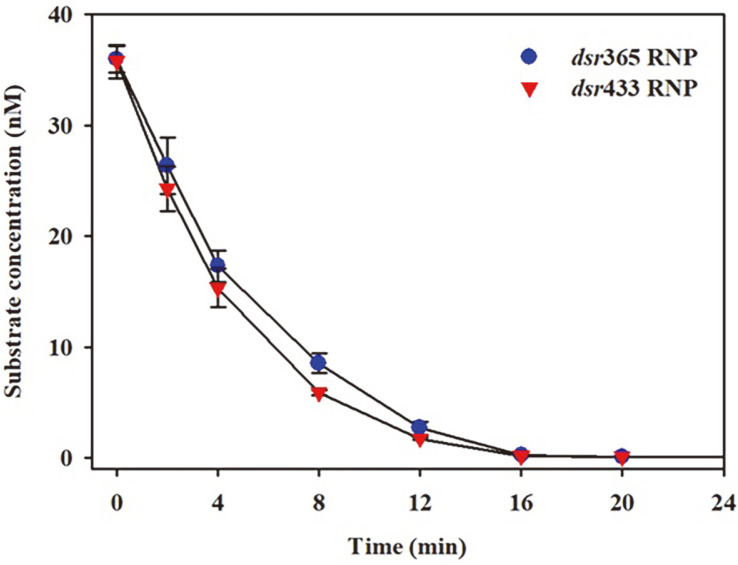
Time courses of DNA double-stranded break of *dsr* substrate by *dsr*365 RNP and *dsr*433 RNP. Measurement of *dsr* concentration was performed after directly mixing *dsr* amplicon and Cas9 RNPs at different time points, by quenching the reaction by heating at 90°C for 5 min. The concentration of *dsr* amplicon was analyzed using the quantitative real-time polymerase chain reaction. Data are the average ± SD of three replicates.

The cleavage reaction of the double-stranded substrate by Cas9 RNP led to a reduction in substrate concentration. As shown in Fig. 6, the *dsr* concentrations were quantified using the qPCR method, revealing a progressive decrease over a 16 min incubation period. This result indicated the complete cleavage of the *dsr* amplicon by both *dsr*365 RNP and *dsr*433 RNP during the reaction. The qPCR results obtained for the cleavage reaction were consistent with those obtained from agarose gel electrophoresis (Fig. 3). When the reaction rates were measured during the initial 2 min, the specific activities of *dsr*365 RNP and *dsr*433 RNP were calculated as 28.74 and 34.48 (unit/μg RNP), respectively. This result revealed that the qPCR assay method accurately assesses the catalytic efficiencies of sgRNAs and Cas9.

**Fig. 7:**
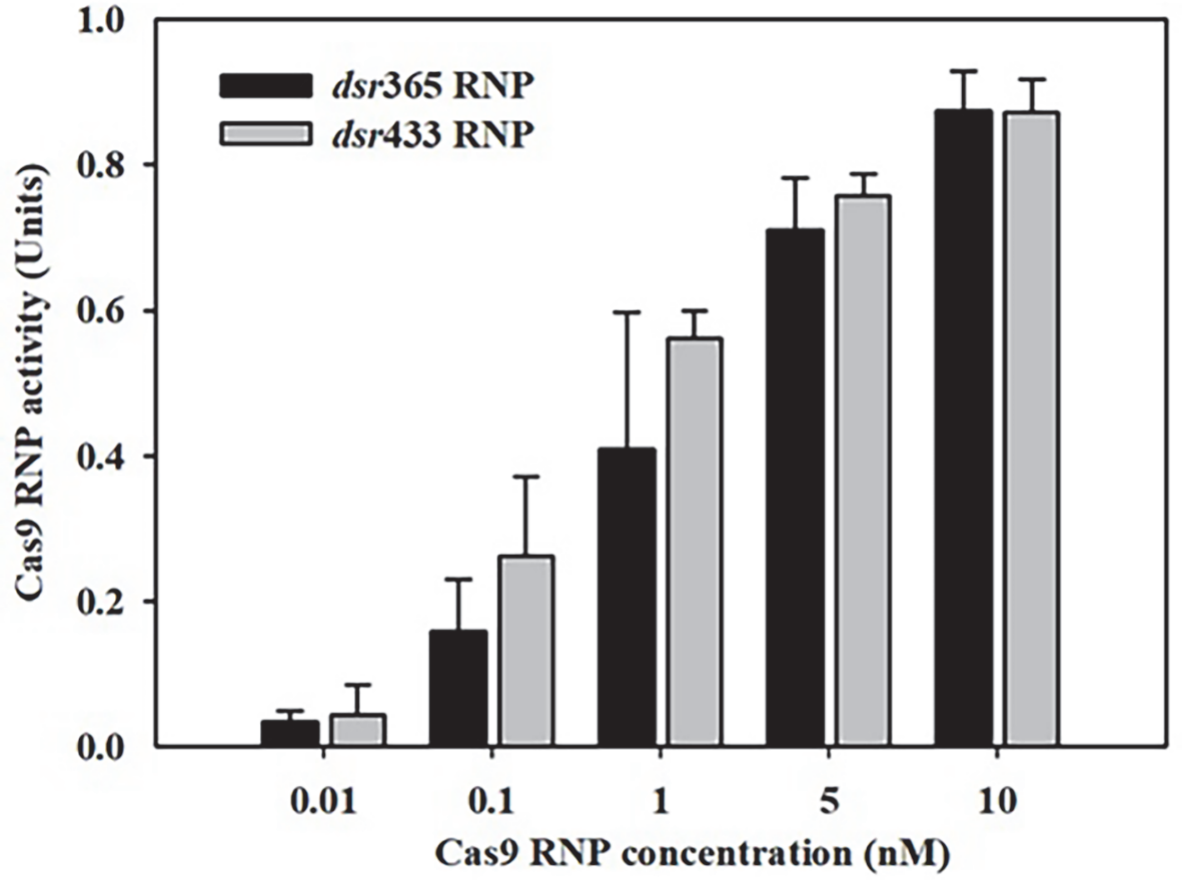
Endonuclease activities of *dsr*365 RNP and *dsr*433 RNP at various concentrations (from 0.01 nM to 10 nM). The Cas9 RNP reaction was performed with 25 nM *dsr* substrate at 37°C for 10 min. The Cas9 RNP reaction was quenched by heating at 90°C for 5 min, and the *dsr* concentrations were analyzed by qPCR. One unit enzyme activity was defined as the amount of enzyme required to cleave one nanomole of *dsr* substrate per minute. Data are the average ± SD of three replicates.

In addition, the Cas9 cleavage reaction was carried out at various RNP concentrations to examine the optimal RNP concentration and monitor the enzyme activity using the qPCR method. As shown in Fig. 7, the Cas9 activities increased along with the increment of RNP concentrations showing their highest values at 10 nM, which was consistent with the findings from agarose gel electrophoresis (Fig. S1). This result exhibited that the qPCR assay method can be applied to measure the cleavage reaction of RNP over a broad concentration range between 0.01 and 10 nM.

To further demonstrate the wide applicability of qPCR method for monitoring Cas9 activity across diverse target DNA, uracil phosphoribosyl transferase (*upp*) gene of *B. bifidum* was used as target DNA for DSB induction using Cas9 RNP. As shown in Fig. S2 the qPCR method effectively tracked the reduction in substrate concentration during the Cas9 reaction using different sgRNAs in *upp* gene. These findings highlight the versatility of the qPCR method in monitoring Cas9 activity across various target DNA sequences.

### Effect of Pulsed Electrical Field on Cas9 RNP Activity

**Fig. 8:**
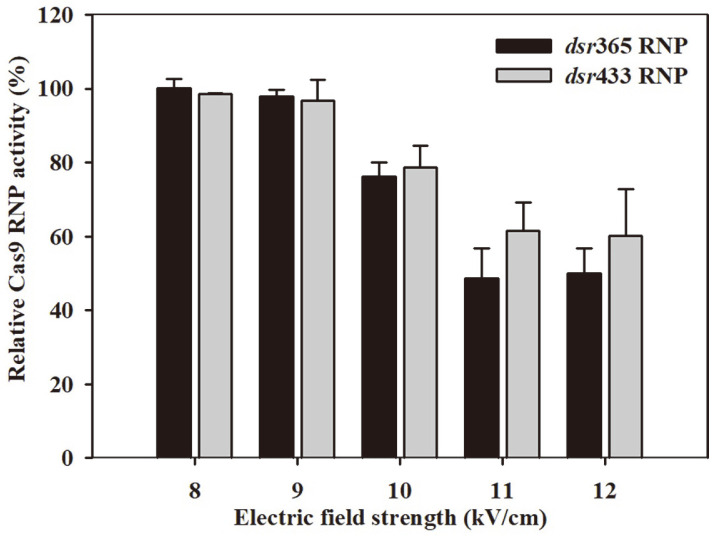
Effect of pulsed electrical field treatment on the endonuclease activities of *dsr*365 RNPs and *dsr*433 RNP. Cas9 complexes were treated with various electrical field strengths (constant 400 Ω and 25 μF), and the remaining activities were measured as described in Methods. The percentage of Cas9 RNP activity was compared to the control (Cas9 RNP without treatment). Data are the average ± SD of three replicates.

The electroporation method, which utilizes high pulsed electrical fields, is widely employed for the physical delivery of Cas9 RNP into cells [29]. To investigate the potential impact of high pulsed electrical fields on the endonuclease activities of Cas9 RNP, the qPCR assay method was utilized to assess their activities following treatment with different electric voltages (8, 9, 10, 11, and 12 kV/cm). As shown in Fig. 8, higher pulsed electrical fields (>10 kV/cm) exhibited detrimental effects on the endonuclease activity of Cas9. Treatment of Cas9 RNP with 8 and 9 kV/cm electrical fields maintained enzyme activity between 96.74% and 100% compared to the control group that did not receive voltage treatment. However, treatment with electrical fields exceeding 10 kV/cm resulted in the loss of enzyme activity, ranging from 21.26% to 51.43%, depending on the strength of the electrical field and the sequence of the sgRNA. This result provided practical insights for successful electroporation delivery of Cas9 RNP to cells, highlighting the importance of maintaining the protein stability of Cas9 at pulsed electrical fields below 10 kV/cm.

## Discussion

Direct delivery of the CRISPR/Cas9 system as a ribonucleoprotein (RNP) complex containing the Cas9 and sgRNA has emerged as a powerful and widespread technique for genome editing due to its short-term genome editing and minimal off-target effects [30]. The endonuclease activity of Cas9 is formed by structural rearrangement during the binding of sgRNA to Cas9 in the presence of metal ions [31]. Therefore, the cleavage efficiency of RNP can be affected by the structure of gRNA [32], the complementarity of genomic sequences [33], the concentration of Mg^2+^ [34], and the stress of electricity. Considering these factors, we developed a qPCR method to assay the endonuclease activity of Cas9-sgRNA RNP complexes and applied it to assay RNP activities at optimal conditions.

A qPCR is a potent tool for identifying and quantifying target DNA in complex DNA samples [20]. Since the outbreak of the COVID-19 pandemic, the qPCR technique has become popular in many laboratories due to its sensitivity and accuracy [35]. This technique has additional advantages, including safety, feasible cost, and short running time. The compounds used in qPCR are primers, dNTP, SYBR green, and dye; thus, it is relatively safe for researchers, while other methods using radioisotopes pose serious health risks [17]. The running time of qPCR was only 1.5–2 h, and the cost of reagents per sample was approximately €1.55, while the electrochemiluminescence method using fluorescence signals needed 4.5 h and €5.15, respectively [36]. In addition, the analyses of Cas9 activity using fluorescence signals such as electro-chemiluminescent [13], fluorescence [14], and microfluidic tool [37] were interfered with by DNA substrate bounded with Cas9 RNP. These methods require the dissociation of DNA substrate from Cas9 RNP complexes by quencher for enzyme assay [37]. In contrast, our new qPCR assay is relatively straightforward compared to the approaches above; it requires quantifying the remaining substrate following the double-stranded break. Moreover, electro-chemiluminescence emission intensities are sensitive to differences in environmental factors, including temperature, solvent, ionic strength, and pH of the analyzed sample [38]. Regarding the advantages of qPCR, our method of assaying Cas9 RNP using qPCR can be commonly utilized with accuracy, cost-benefit, and safety.

The measurement of specific activities for the RNPs in our study revealed that *dsr*433 RNP exhibited a higher value (34.48 units/μg RNP) compared to *dsr*365 RNP (28.74 units/μg RNP). This result indicated that our method enables accurate measurement of catalytic efficiencies between different Cas9 RNP. The observed disparity in specific activities between the RNP raises two potential explanations. Firstly, the endonuclease activity of Cas9 RNP is known to be formed through structural rearrangement during the binding of sgRNA to Cas9 in the presence of metal ions [31]. Consequently, the way in which sgRNA activates the endonuclease function of Cas9 can vary. Secondly, it is well established that sgRNA can influence the DNA binding affinity of Cas9 RNP, thereby impacting Cas9 activity [37]. The variations in specific activities between the RNPs may be attributed to the divergent effects of sgRNA on the DNA binding affinity with Cas9 RNP. The specific sequence and structure of the sgRNA molecules may modulate their interaction with Cas9, resulting in distinct outcomes in terms of Cas9 activity. Overall, our findings suggest that the specific activities of Cas9 RNP can be influenced by the interplay between sgRNA, Cas9, and the target DNA.

By using a qPCR-based method, the activity of Cas9 RNP was measured from a concentration of 0.01 nM to 10 nM Cas9 RNP (Fig. 7), and the limit of detection (LOD) for the Cas9 RNP concentration was 0.01 nM. Our method has a lower LOD compared to other methods that use electrochemiluminescence, which has an LOD of approximately 1 nM [13]. The enhanced sensitivity of our qPCR-based method can be attributed to the catalytic mechanism of Cas9. The Cas9 from *S. pyogenes* is recognized as a single-turnover enzyme, leading to a gradual degradation and slow release of the product following substrate binding [40]. This feature presents a challenge for accurate analysis of enzyme activity using fluorescence-based signals such as electrochemiluminescence [13], fluorescence [14], and microfluidic tools [37]. These methodologies require the dissociation of the DNA substrate from Cas9 RNP complexes in order to release the signal from the quencher. In contrast, our qPCR-based method overcomes this limitation as it solely necessitates the quantification of the remaining substrate subsequent to the occurrence of double-stranded breaks, even if the Cas9 RNP does not dissociate from the product. The improved LOD and the ability to bypass the requirement for dissociation of Cas9 RNP from the product render our qPCR-based method advantageous for measuring Cas9 activity. This approach provides a robust and sensitive means to assess Cas9-mediated cleavage, facilitating accurate characterization of enzyme efficiency and enabling its application in various research and clinical contexts.

Nevertheless, it is important to acknowledge the limitations of our qPCR-based method for measuring the endonuclease activity of Cas9 RNP. One limitation arises from the observed non-linear curve of Cas9 RNP activity during the incubation period (Fig. 6). This non-linearity can be attributed to the prolonged binding of the Cas9 enzyme to the targeted DNA following double-stranded break, resulting in slow product release [41]. Consequently, the enzymatic activity of Cas9 deviates from the conventional Michaelis-Menten model [37]. In our study, we addressed this challenge by calculating the reaction rate of Cas9 based on the measurement of DNA concentration within a feasible time frame of 2 min using the qPCR method. However, it should be noted that our approach did not allow for continuous monitoring of DNA concentration changes, which is still possible with alternative methods such as radioactive signals, fluorescence, and microfluidic devices. Continuous monitoring would offer a more comprehensive understanding of the temporal dynamics of the Cas9 cleavage reaction. To overcome this limitation, an alternative strategy could involve measuring DNA concentration at shorter intervals. By reducing the time intervals between measurements, it may be possible to capture a more dynamic representation of Cas9 activity, providing insights into the kinetics of the enzymatic process. This approach would allow for a more accurate assessment of the temporal changes in DNA concentration and potentially provide a more comprehensive characterization of Cas9 activity. Future improvements in the methodology could focus on refining the temporal resolution of DNA concentration measurements to enhance our understanding of Cas9 kinetics and to further optimize the accuracy and reliability of our qPCR-based method for assessing Cas9 activity.

In conclusion, our study presents a novel qPCR-based method for monitoring Cas9 endonuclease activity. This approach offers simplicity, convenience, safety, and stability. Therefore, it provides a valuable tool for comparing Cas9 activity using different sgRNAs in genome editing of prokaryotic and eukaryotic organisms. The wide applicability of this qPCR-based assay method makes it a promising asset for the advancement of Cas9 RNP-mediated genome editing in various domains, including humans, animals, plants, and microorganisms.

## Supplemental Materials

Supplementary data for this paper are available on-line only at http://jmb.or.kr.

## Figures and Tables

**Table 1:** Oligonucleotides sequence used in this study.

Name	Primer sequence (5’ → 3’)	GC content (%)	Base pair	T_m_ (°C)	Target
DSU-F	TAGGCATGTTGTATTGTGTATATTTC	30	27	58	*dsr* amplicon
DSU-R	AACTAATCAACCACGTCGCACCATA	45	25	58	
UPP-F	TGCCCGCGTTGTATTTCGAGGT	44	22	59	*upp* amplicon
UPP-R	GCCACCATGCAACCAGCGAATC	49	22	59	
q-DSU-F	TTAGCAAGTGTGTTATCACTGGTTAC	38	26	58	qPCR
q-DSU-R	AGCATCAGACACAACGACTGATA	43	23	57.5	
q-UPP-F	ATCATTTTGGTCATGCCGTCG	48	21	58	
q-UPP-R	ACTCGTCATGCTCGAAGCCTA	52	21	58	
F(DS365)	GAAATTAATACGACTCACTATAGCGTAACAGTCG GCAACTGCTGTTTTAGAGCTAGAAATAGCAAG	39	66	69	Transcription sgRNA
F(DS433)	GAAATTAATACGACTCACTATAGATCTGGAAAAA GATGGTAAAGTTTTAGAGCTAGAAATAGCAAG	32	66	66	
F(UPP182)	GAAATTAATACGACTCACTATAGCGAAACCCCCG TCGCCCCCAGTTTTAGAGCTAGAAATAGCAAG	45	66	71	
F(UPP212)	GAAATTAATACGACTCACTATAGCGAAGGACGGG AACGATGAGTTTTAGAGCTAGAAATAGCAAG	40	65	69	
R(common)	AAAAAAGCACCGACTCGGTGCCACTTTTTCAAG TTGATAACGGACTAGCCTTATTTTAACTTGCTAT TTCTAGCTCTAAAAC	38	82	71	

**Table 2:** Prediction of sgRNA sequence candidates targeting dextransucrase gene (*dsr*) using CRISPOR database.

Name	Guide sequence (5’ → 3’) PAM	Predicted efficiency (%)
DS365	CGTAACAGTCGGCAACTGCT *TGG*	81
DS376	TTAGCTGTTGGCGTAACAGT *CGG*	67
DS388	CCAGTTTTTTGATTAGCTGT *TGG*	78
DS433	ATCTGGAAAAAGATGGTAAA *TGG*	87
UPP182	CGAAACCCCCGTCGCCCCCA *TGG*	71
UPP212	GCGAAGGACGGGAACGATGA *TGG*	73
