# Supplementary material for: A qPCR Method to Assay Endonuclease Activity of Cas9-sgRNA Ribonucleoprotein Complexes
Source: J Microbiol Biotechnol. 2023 Jun 19;33(9):1228–37. doi: 10.4014/jmb.2305.05010 (PMC10580886; doi:10.4014/jmb.2305.05010)
Supplement: Supplementary file 1 [file jmb-33-9-1228-supple.pdf]

Supplementary Figure

A qPCR Method to Assay Endonuclease Activity of Cas9-sgRNA Ribonucleoprotein Complexes

Minh Tri Nguyen[1,4], Seul-Ah Kim[1], Ya-Yun Cheng[1], Sung Hoon Hong[1], Yong-Su Jin[2,3], and Nam Soo Han[1*]

[1] Brain Korea 21 Center for Bio-Health Industry, Division of Animal, Horticultural, and Food Science, Chungbuk National University, Cheongju, 28644, Republic of Korea

[2] Department of Food Science and Human Nutrition, University of Illinois at Urbana-Champaign, Urbana, IL 61801, USA

[3] Carl R. Woese Institute for Genomic Biology, University of Illinois at Urbana-Champaign, Urbana, IL 61801, USA

[4] Faculty of Biology, Dalat University, 01- Phu Dong Thien Vuong, Dalat, Vietnam

***Corresponding author:**

Prof. Nam Soo Han (ORCID: 0000-0002-8460-8165)

Phone: +82-43-261-2567, Fax: +82-43-271-4412;

E-mail: namsoo@cbnu.ac.kr

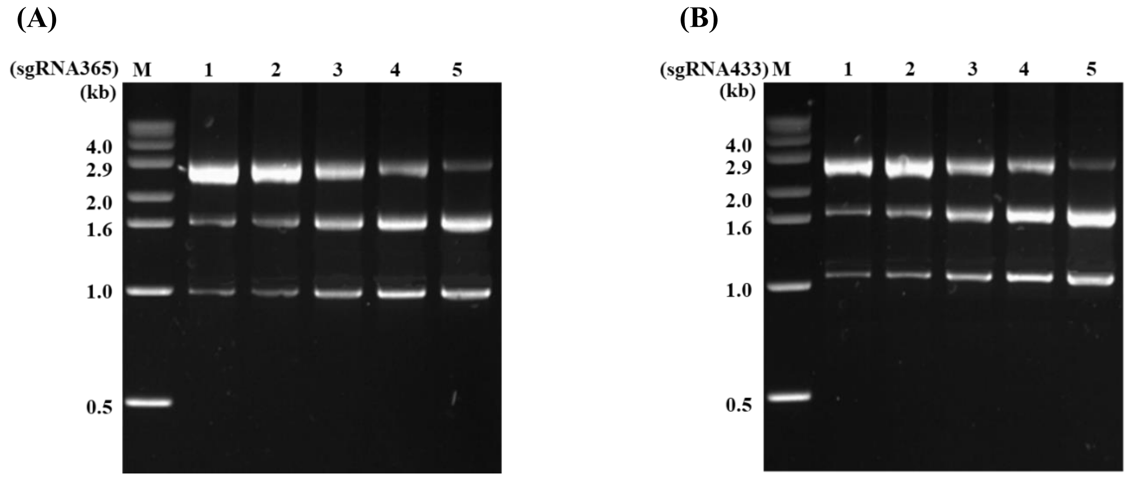

Fig. S1. Agarose gel electrophoresis of *dsr*365 RNP and *dsr*433 RNP at various concentrations. The Cas9 RNP reaction was performed with *dsr* substrate at 37°C for 10 min. The Cas9 RNP reaction was quenched by heating at 90°C for 5 min, and the *dsr* concentrations were analyzed by gel electrophoresis. Lane M; marker for 1kb, Lane 1; 0.01 nM RNP, Lane 2; 0.1 nM RNP, Lane 3; 1 nM RNP, Lane 4; 5 nM RNP, Lane 5; 10 nM RNP.

(A) (B)

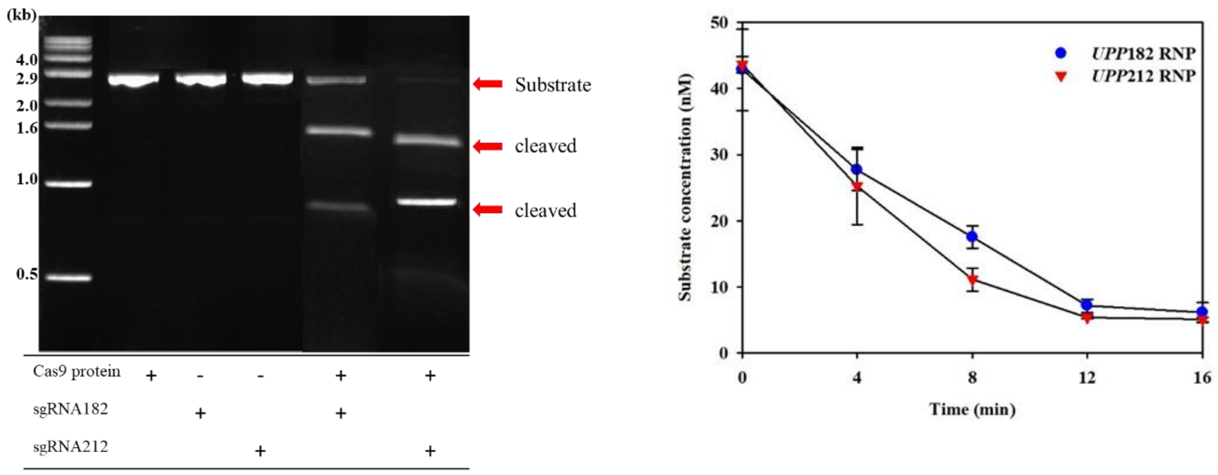

Fig. S2. *In vitro* double-stranded breaks in *upp* amplicon by *UPP*182 RNP and *UPP*212 RNP (A). Time courses of DNA double-stranded break of *upp* substrate by *UPP*182 RNP and *UPP*212 RNP. Measurement of *upp* concentration was performed after directly mixing *upp* amplicon and Cas9 RNPs at different time points, by quenching the reaction by heating at 90 ^{o}C for 5 min. The concentration of *upp* amplicon was analyzed using the quantitative real-time polymerase chain reaction (B). Data are the average ± SD of three replicates.
